# Fragment Library of Natural Products and Compound Databases for Drug Discovery †

**DOI:** 10.3390/biom10111518

**Published:** 2020-11-06

**Authors:** Ana L. Chávez-Hernández, Norberto Sánchez-Cruz, José L. Medina-Franco

**Affiliations:** DIFACQUIM Research Group, Department of Pharmacy, School of Chemistry, Universidad Nacional Autónoma de México, Avenida Universidad 3000, Mexico City 04510, Mexico; anachavez3026@gmail.com (A.L.C.-H.); norberto.sc90@gmail.com (N.S.-C.)

**Keywords:** chemoinformatics, COVID-19, drug discovery, drug design, fingerprint, food chemicals, natural products fragments, SARS-CoV-2

## Abstract

Natural products and semi-synthetic compounds continue to be a significant source of drug candidates for a broad range of diseases, including coronavirus disease 2019 (COVID-19), which is causing the current pandemic. Besides being attractive sources of bioactive compounds for further development or optimization, natural products are excellent substrates of unique substructures for fragment-based drug discovery. To this end, fragment libraries should be incorporated into automated drug design pipelines. However, public fragment libraries based on extensive collections of natural products are still limited. Herein, we report the generation and analysis of a fragment library of natural products derived from a database with more than 400,000 compounds. We also report fragment libraries of a large food chemical database and other compound datasets of interest in drug discovery, including compound libraries relevant for COVID-19 drug discovery. The fragment libraries were characterized in terms of content and diversity.

## 1. Introduction

Natural products (NP) have long been studied and used in medicine and chemistry, starting from ancient civilizations throughout history. Natural sources were the basis of early research in medicinal chemistry and drug discovery and have yielded valuable therapeutic agents still in use today [1]. A recent review reveals that 3.8% of drugs approved between 1981 and 2019 are NP, and 18.9% are NP derivatives [2].

The unique and complex chemical structures of NP make them unique sources to explore novel areas of the chemical space [3]. However, considering the structural complexity of NP, it is a challenge to produce them in large quantities, which is typically required during drug development. Therefore, in recent years novel methods and synthetic strategies have been developed to obtain diverse and semi-synthetic compounds libraries based on NP [4]. Similarly, NP are becoming attractive starting points to conduct fragment-based drug design and build the so-called “pseudo-NPs” [5].

The increasing use of NP in modern drug discovery has promoted the application of chemoinformatic methods for natural product-based drug discovery. One such contribution is the generation and development of compound databases [6,7,8]. The development of compound databases of NP and synthetic analogs has been recently reviewed [8,9]. A recent notable example is the COlleCtion of Open NatUral producTs (COCONUT), a compendium of 50 open-access databases collecting more than 400,000 compounds. These and other public collections of food chemicals are important sources to generate fragment libraries of compounds of natural origin. The authors recently reported and made public a library with 205,903 fragments derived from a drug-like subset of the first version of COCONUT [10]. In that work, a total of 190,139 molecules were analyzed. Recently COCONUT was updated, and a fragment library based on its full comprehensive collection has not been reported.

The goal of this work was to generate a fragment library of the complete and most recent version of COCONUT that contains 432,706 compounds. We also expanded the analysis to generate fragment libraries of large public collections of 23,883 food chemicals that have a close association with NP [11] and are part of the increasing research field of *foodinformatics* [12]. The fragment libraries were characterized using chemoinformatic methods and compared with reference fragment libraries generated from molecules in the Dark Chemical Matter (DCM). DCM is a collection of 139,352 compounds that showed no activity when tested in at least 100 screening assays but that have recently led to the identification of bioactive compounds [13]. In light of the current coronavirus disease 2019 (COVID-19) pandemic, we also included in this study two large reference libraries with relevance in drug discovery in relation to this disease [14]. Of note, food chemicals and DCM compounds analyzed in this work were recently screened in silico to identify potential inhibitors of the main protease of severe acute respiratory syndrome coronavirus 2 (SARS-CoV-2), one of the main promising molecular targets for the treatment of COVID-19 [15].

## 2. Materials and Methods

### 2.1. Compound Databases

In this work, we generated fragment libraries of five compound databases of interest in drug discovery, summarized in Table 1 and listed here: COCONUT, the largest database, with a total of 423,706 unique molecules [16], Food Database (FooDB) with 23,883 food chemicals [17], and a database with 139,352 small molecules, classified as DCM [13]. We also analyzed a focused public library relevant to COVID-19 research assembled by the Chemical Abstract Service (CAS) with 48,876 compounds [18] and 280 inhibitors of the main protease of SARS-CoV-2 (3CLP) [15].

### 2.2. Data Curation

Similar to our previous work [10], the preparation of the five datasets was performed with the open-source cheminformatics toolkit RDKit [19], (version 2020.03.2.0, RDKit, San Francisco, CA, USA) and the functions Standardizer, LargestFragmentChoser, Uncharger, Reionizer, and TautomerCanonicalizer implemented in the molecule validation and standardization tool MolVS [20]. SMILES strings [21], with no stereochemistry information, were generated because not all compounds in the datasets have a defined stereochemistry. Compounds with valence errors or any chemical element other than H, B, C, N, O, F, Si, P, S, Cl, Se, Br, and I were removed. With the chemical compounds retained, neutralized, and reionized, a canonical tautomer was generated. The average molecular weight (AMW) was calculated, and all compounds with AMW ≤ 1300 were retained. Table 1 summarizes the number of compounds used for the fragmentation analysis and the number of unique fragments generated.

### 2.3. Generation of Unique Fragments Using the RECAP Algorithm

Fragment libraries were produced with the Retrosynthetic Combinatorial Analysis Procedure (RECAP) as implemented in RDKit (version 2020.03.2.0, RDKit, San Francisco, LA, USA). The RECAP algorithm is based on 11 cleavage rules derived from chemical reactions [22]. A molecule is cleaved into fragments if it contains any of the following bonds: amide, ester, amine, urea, ether, olefin, quaternary nitrogen, aromatic nitrogen–aliphatic carbon, lactam nitrogen–aliphatic carbon, aromatics carbon–aromatic carbon, and sulphonamide. For this study, only terminal fragments were generated.

All curated datasets and fragments libraries used in this work are available at https://doi.org/10.6084/m9.figshare.13064231.v1. Datasets contain the curated structures and the following information: identification number (ID), simplified molecular input line entry system (Smiles), Average Molecular Weight (AMW), number of carbons, oxygens, nitrogens, heavy atoms, aliphatic rings, aromatic rings, heterocycles and bridgehead atoms, fraction of sp^3^ carbon atoms and chiral carbons, and a list of fragments generated from each compound. Fragment libraries contain structures generated (Fragments) from each compound library (Dataset) and the following information: number of compounds that contain that fragment in a dataset (Count) and fraction of them (Proportion), Average Molecular Weight (AMW), number of carbons, oxygens, nitrogens, heavy atoms, aliphatic rings, aromatic rings, heterocycles and bridgehead atoms, fraction of sp^3^ carbon atoms and chiral carbons.

### 2.4. Structural Diversity and Complexity

The structural diversity of the compounds and fragment datasets was evaluated by calculating the median value of the distribution of the pairwise similarity values generated with the Tanimoto coefficient for both Morgan fingerprint with radius 2 (Morgan2, 1024-bits) [23] and Molecular ACCes System (MACCS) keys (166-bits) [24]. For 4 sets of entire compounds (except 3CLP), the calculation was done for 10 random samples of 10,000 compounds each, and the medians were then averaged. For 3CLP, all 256 molecules were used. For the fragment datasets, all fragments were employed for the calculation, except for COCONUT, for which 10 random samples of 10,000 fragments were used. It has been shown that for large datasets, several random samples of 1000 compounds each are a reasonable approach to quantify the pairwise fingerprint-based diversity of the entire datasets [25].

The structural differences between compound and fragment datasets were evaluated, calculating 14 molecular descriptors, namely, number of carbon, oxygen, nitrogen, and heavy atoms, the number of rings and heterocycles—both aliphatic and aromatic—spiro atoms, bridgehead atoms, the fraction of sp^3^ carbons, and chiral carbons.

### 2.5. Chemical Space Visualization

Morgan fingerprints with radius 2 (Morgan2, 1024-bits) were generated for each compound and fragment data set. To generate a visual representation of the chemical space, we used the recently developed algorithm TMAP (Tree MAP). This method allows the visual representation of many molecules that are difficult to visualize using other standard methods such as principal component analysis. Basically, TMAP allows the visualization of large data sets (such as the ones studied in this work—Table 1) through the distance between the clusters and the cluster’s detailed structure through branches and sub-branches [26,27]. Fingerprints for each data set (input data) were indexed in a local sensitive hashing (LSH) forest data structure, enabling c-approximate k-nearest neighbor (k-NN). Fingerprints were encoded using the MinHash algorithm. An undirected weighted c-approximate k-nearest neighbor graph (c-k-NNG) is constructed from the data points indexed in the LSH forest. This graph takes two arguments, k, the number of nearest-neighbors, and kc, the factor used by the augmented query algorithm. In this work, we used k = 50 and kc =10. Further details of the TMAP approach are published elsewhere [28].

## 3. Results and Discussion

### 3.1. Overlapping Fragments and Compounds

**Figure 1:**
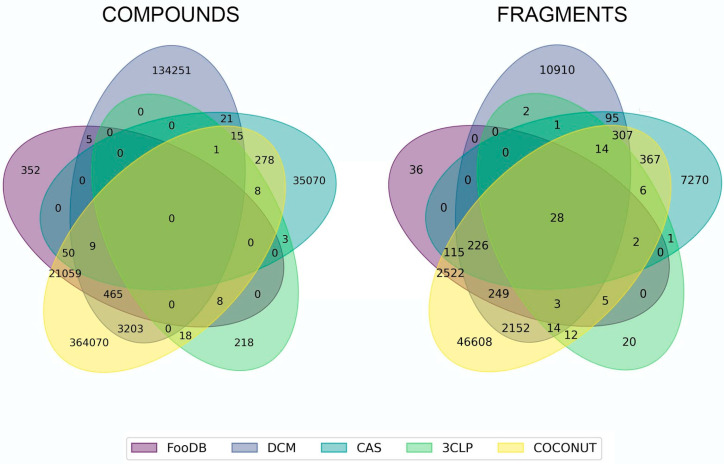
Unique and overlapping compounds and fragments from COCONUT, FooDB, DCM, CAS, and 3CLP. Compounds and fragments are represented with colors: yellow (COCONUT), violet (FooDB), purple (DCM), green (CAS), and lime (3CLP).

Figure 1 shows the number of unique and overlapping compounds and fragments. We found 533,961 unique compounds among all datasets which comprising 364,070 COCONUT compounds (93.54%), 352 from FooDB (1.6%), 134,251 from DCM (96.35%), 35,070 from CAS (78.31%), and 218 from 3CLP (85.15%). The largest compound overlap occurred between COCONUT and FooDB (21,591 (98.37%) FooDB compounds in COCONUT). The second largest overlap was between COCONUT and 3CLP (concerning 35 (13.67%) 3CLP compounds), followed by the overlaps between COCONUT and DCM (concerning 3693 (2.65%) DCM compounds) and COCONUT and CAS (concerning 361 (0.26%) CAS compounds).

Regarding the fragments, Figure 1 indicates that there we identified 64,844 unique fragments among all datasets, including 46,608 COCONUT fragments, 36 FooDB fragments (1.12%), 10,910 DCM fragments (77.92%), 7270 CAS fragments (86.21%), and 20 3CLP fragments (18.51%). The largest fragment overlap occurred for 3150 FooDB fragments (98.87%) overlapped with COCONUT fragments, followed by 84 3CLP fragments (77.77%), 2993 DCM fragments, and 1065 CAS fragments. We also found that 28 fragments were shared by all fragment libraries (Figure 1).

It should be noted that around 13% of 3CLP inhibitors are found within a global dataset of NP. Likewise, 77% of 3CLP fragments can be obtained from NP. This observation reinforces our hypothesis that previously isolated and characterized NP are potential sources of compounds against COVID-19. In turn, this is also in agreement with several reports of virtual screenings of NP databases aimed to identify compounds with activity against 3CLP [29,30].

### 3.2. Fragment Analysis

**Figure 2:**
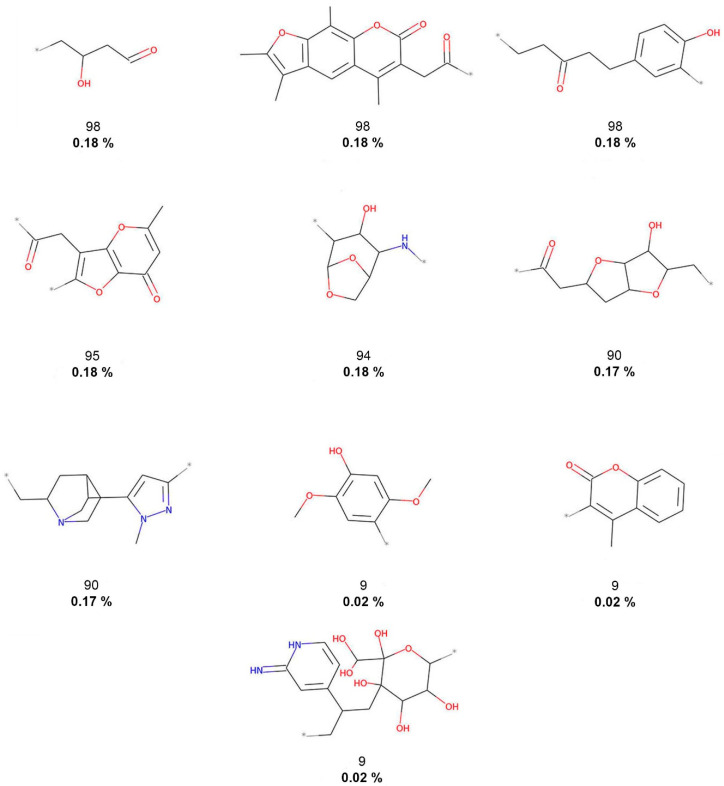
The 10 most frequent and unique COCONUT fragments. Frequency (regular font) and proportion (bold font) are listed below the chemical structures.

**Figure 3:**
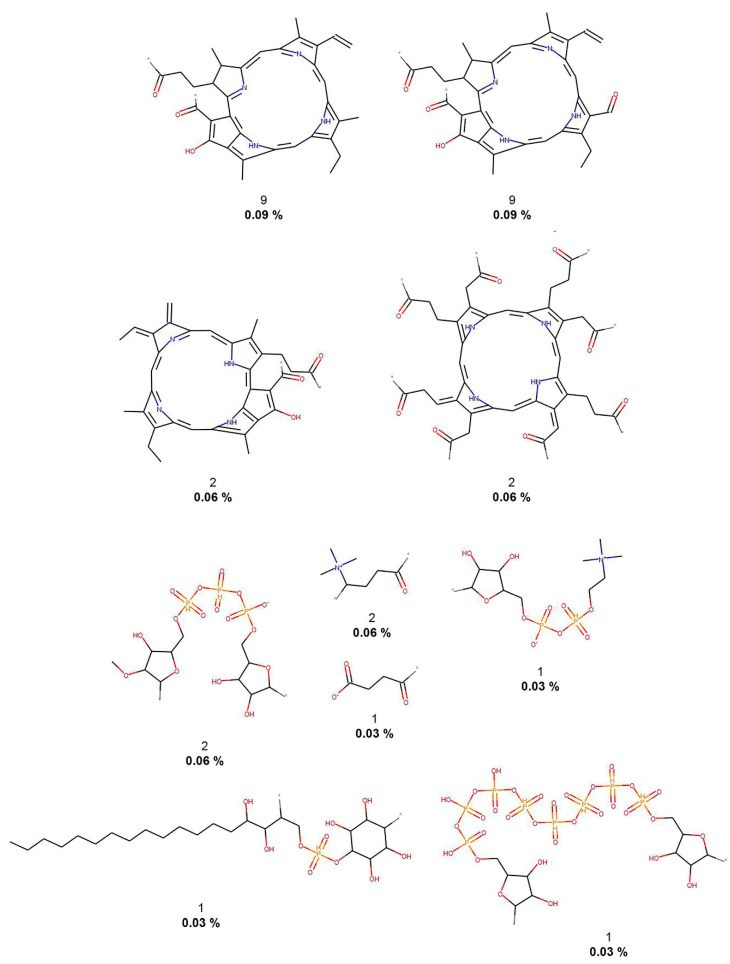
The 10 most frequent and unique FooDB fragments. Frequency (regular font) and proportion (bold font) are listed below the chemical structures.

**Figure 4:**
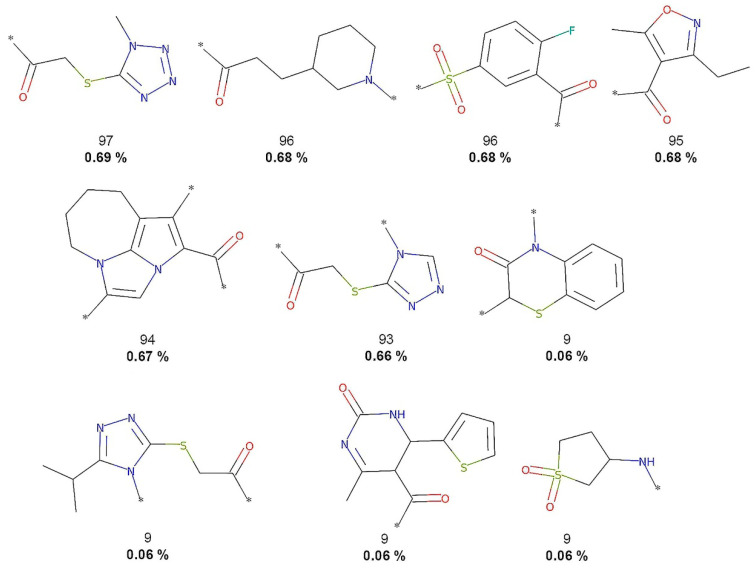
The 10 most frequent and unique DCM fragments. Frequency (regular font) and proportion (bold font) are listed below the chemical structures.

**Figure 5:**
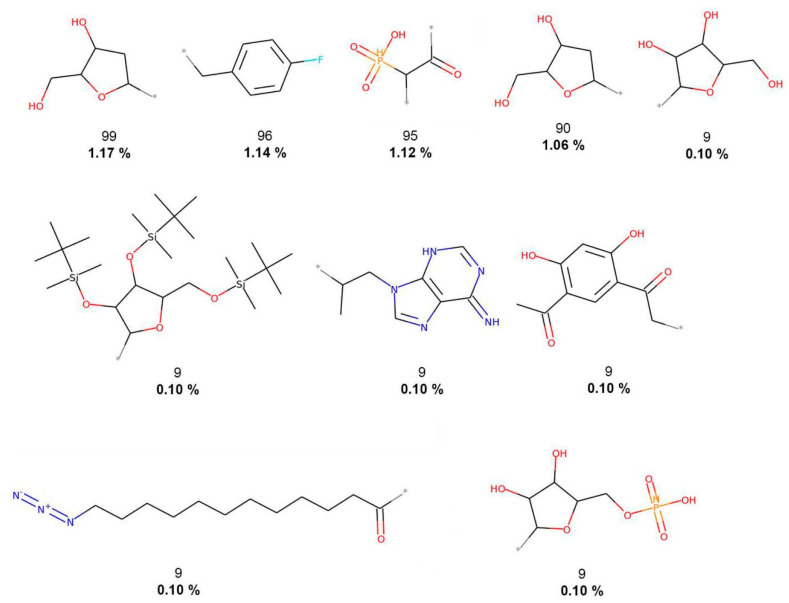
The 10 most frequent and unique CAS fragments. Frequency (regular bond) and proportion (bold font) are listed below the chemical structures.

**Figure 6:**
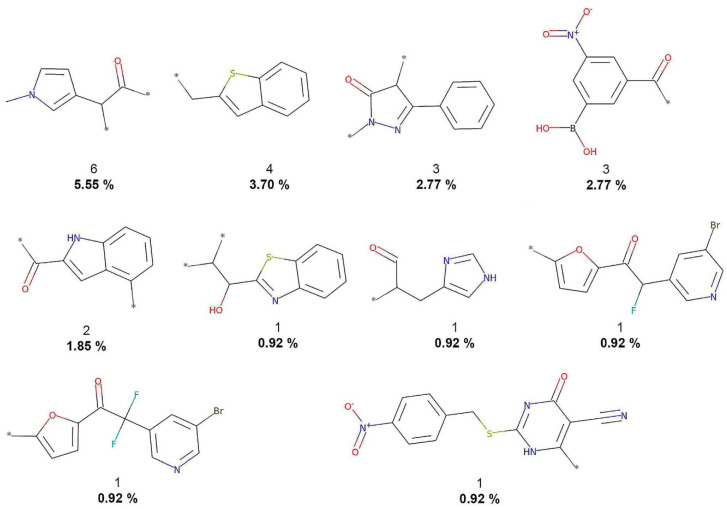
The 10 most frequent and unique 3CLP fragments. Frequency (regular bond) and proportion (bold font) are listed below the chemical structures.

As described in the Methods Section 2.3, molecular fragments (terminal fragments only) were obtained from the five compound datasets. The NP fragments in COCONUT and the food chemicals in FooDB were compared with molecules of three reference datasets: small molecules with no biological activity despite having been exhaustively tested in high-throughput screening (HTS) and two collections for COVID-19 drug discovery. Table 1 summarizes the results. The largest number of different fragments was generated for COCONUT (52,630), while the smallest number of fragments was calculated for 3CLP (108). Figure 2, Figure 3, Figure 4, Figure 5 and Figure 6 show the chemical structures of the 10 most frequent and unique fragments in the 5 databases studied. The figure indicates the frequency and percentage of each fragment in the corresponding dataset.

Figure 2 shows that COCONUT fragments contain the largest number of oxygen atoms (carbonyls, alcohols, and aldehydes), aliphatic rings, like tetrahydrofurans and pyranones, and other oxygen-containing heterocycles. FooDB fragments are characterized by having macrocycles (porphyrin rings) and triphosphates groups (Figure 3). In contrast, fragments from CAS, 3CLP, and DCM have larger numbers of nitrogen atoms and aromatic rings than fragments from COCONUT and FooDB as shown in Figure 4, Figure 5 and Figure 6. The most frequent DCM fragments contain various triazole and pyrimidine rings, and 3CLP fragments comprise pyrrole, imidazole, and pyrazole rings.

**Figure 7:**
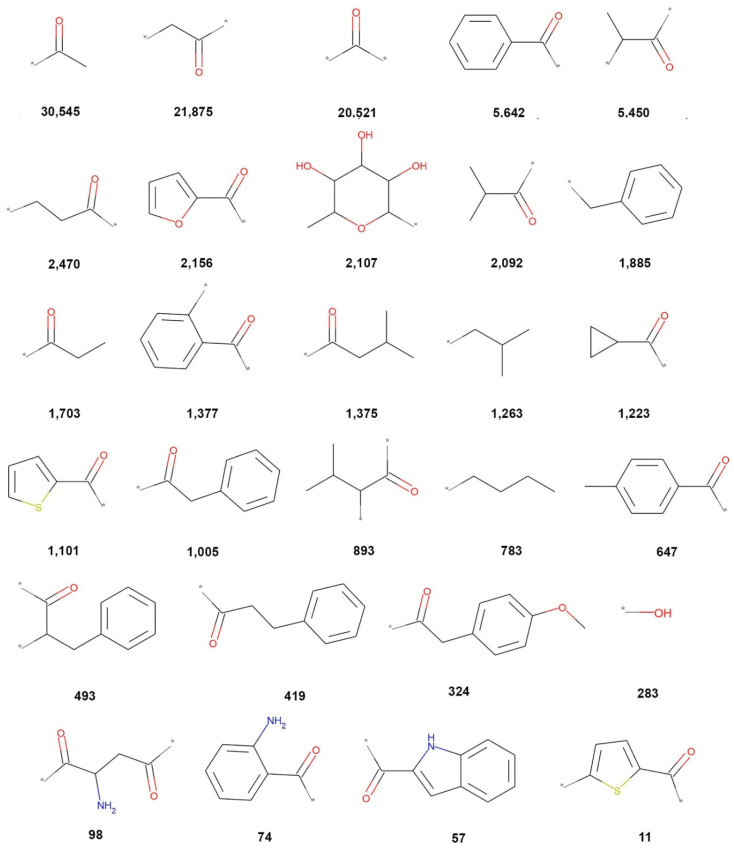
Overlapping fragments between COCONUT, FooDB, DCM, CAS, and 3CLP. The sum of frequencies of each fragment in all databases is indicated in bold font.

The chemical structures of the 28 fragments common (overlap) to all five data sets (Figure 1) are represented in Figure 7, which shows the sum of frequencies of each fragment in all databases and the cleavage bonds in gray color (also marked with *). Relevant overlapping fragments include acetophenones (5642, 1377, and 647), 2-acetylfuran (2156), cyclopropyl methyl ketone (12,223), benzylacetone (493, 419), 2-acetylthiophene (1101 and 11), 2-aminohexane-2,5-dione (98), 2-aminoacetophenone (74), 2-acetylindole (57).

Table 2 and Table 3 summarize the distribution of carbon, oxygen, nitrogen, and heavy atoms for the entire compounds and fragment datasets, respectively. The tables also summarize the fraction of sp^3^ carbon atoms and chiral carbons as representative structural complexity measures. Finally, both tables indicate the distribution of the number of rings (total number, aliphatic, and aromatic) and other important structural features of the compound and fragment datasets. Table 2 shows that compounds from COCONUT and FooDB have the highest mean fraction of sp^3^ carbons, 0.506 and 0.620, respectively, whose values range from 0.45 and 0.59 for NPs [31]. CAS, DCM, and 3CLP show the largest number of aromatic rings and aromatic heterocycles, which are characteristic of drugs and synthetic compounds [32]. Compounds in COCONUT and FooDB have the largest number of carbon and oxygen atoms, fraction of chiral carbons, and number of aliphatic rings and bridgehead atoms, a trend that is preserved for their respective fragments (see Table 3). However, fragments from COCONUT and FooDB overlapping with those from CAS, DCM, and 3CLP have the lowest number of carbon, oxygen, and aliphatic rings, compared to unique fragments (Table 3).

In general, NPs have been reported to have a higher fraction of sp^3^ carbons (associated with a greater structural complexity) and number of oxygen atoms and a lower number of nitrogen atoms and aromatics rings as well as NP fragments [31,33]. Therefore, the fragments from COCONUT and FooDB are also attractive as building blocks for designing drug candidates. 

### 3.3. Structural Diversity and Complexity

The fingerprint-based structural diversity was measured as the median value of the distribution of the pairwise similarity values calculated with the Tanimoto Coefficient, both MACCS keys and Morgan2 (see Methods, Section 2.4). The results are summarized in Table 4 and Table 5. Regarding the diversity of the compound libraries, FooDB was the most diverse in terms of Morgan2 and MACCS keys fingerprints (median similarity of 0.092, 0.322), followed by COCONUT (0.107, 0.380) (Table 4). The structural diversity of the most recent version of COCONUT (studied in this work) is similar to the fingerprint diversity calculated for a drug-like subset of COCONUT (0.117, 0.314) computed recently [10]. CAS appeared to be one of the least diverse sets, which is consistent because the datasets were selected by focusing on COVID-19 research (vide supra).

Regarding the fingerprint-based diversity of the fragment datasets (Table 5), in general, all fragment libraries showed a larger diversity than their parent compounds. Specifically, the CAS fragments were the most diverse according to both molecular fingerprints (0.094, 0.222), followed by FooDB (0.106, 0.241) and COCONUT (0.111 only for Morgan2). Possibly, the difference in the diversity of the fragments from NP in COCONUT and food chemicals in FooDB is associated with the fragmentation algorithm (i.e., the RECAP fragmentation algorithm terminal fragments only as compared to our previous work [10]). This result means that the diversity of fragments appears in the intermediate compounds generated throughout the fragmentation process.

### 3.4. Chemical Space Visualization

**Figure 8:**
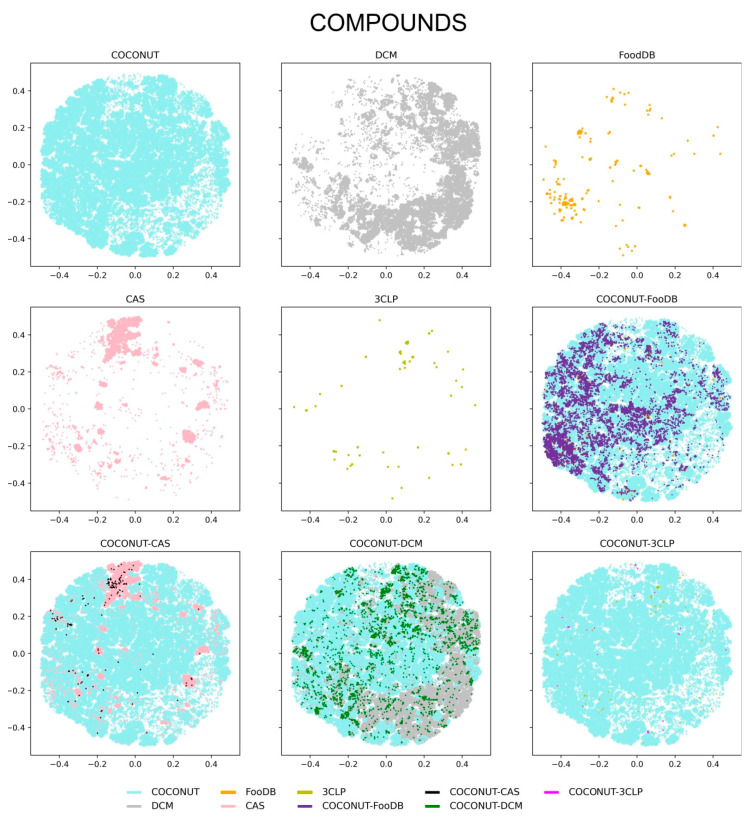
Visualization of the chemical space of the compound datasets generated with Tree Maps. Datasets are represented with colors: COCONUT (cyan), DCM (gray), FooDB (orange), CAS (pink), and inhibitors of the main protease of SARS-CoV-2, 3CLP, (olive). Overlapping compounds in COCONUT–FooDB (purple), COCONUT–CAS (black), COCONUT–DCM (green), and COCONUT–3CLP (magenta) are indicated.

**Figure 9:**
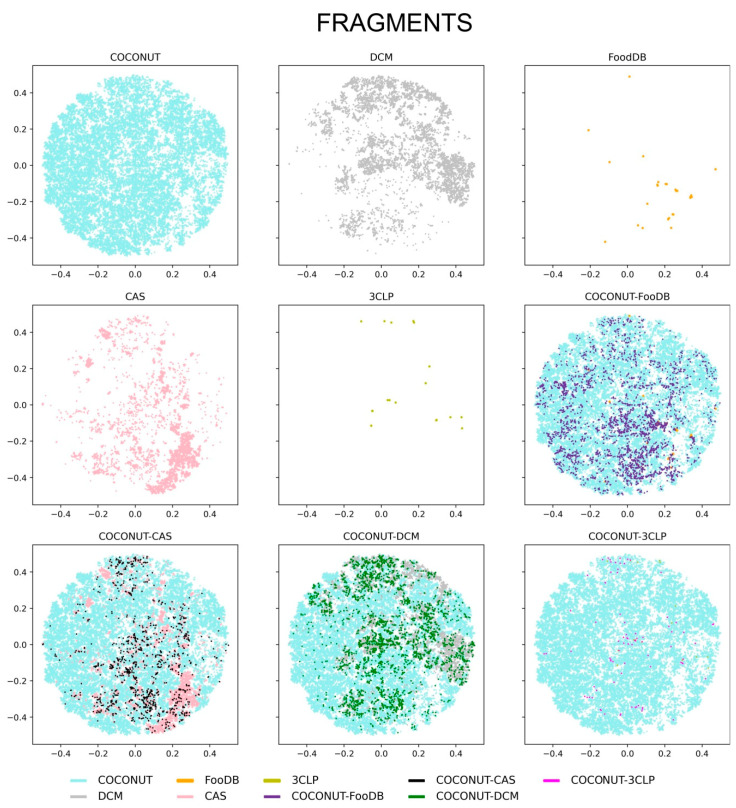
Visualization of the chemical space of fragments generated with Tree Maps. Datasets are represented with colors: COCONUT (cyan), DCM (gray), FooDB (orange), CAS (pink), and inhibitors of the main protease of SARS-CoV-2, 3CLP, (olive). Overlapping fragments in COCONUT–FooDB (purple), COCONUT–CAS (black), COCONUT–DCM (green), and COCONUT–3CLP (magenta) are indicated.

A visual representation of the chemical space of the entire compounds and fragments was explored using the TMAP approach, as described in Methods, Section 2.5. Of note, TMAPs facilitate the visualization of very large datasets (e.g., more than 380,000 molecules from COCONUT, Table 1). The visual representation of the chemical space for the entire compounds and fragments is shown in Figure 8 and Figure 9, respectively. The figures display the chemical space of all compounds and fragments using the same coordinates. To improve the visualization’s clarity, each set of unique compounds and fragments from the five datasets is shown individually. The figures also present three panels showing direct comparisons of COCONUT with the other datasets, highlighting in different colors the compounds that are in common, i.e., COCONUT–FooDB (purple); COCONUT–CAS (black); COCONUT–DCM (green), and COCONUT–3CLP (magenta).

Figure 8 shows that all compound datasets converged in the chemical space largely defined by COCONUT, followed by that of DCM. The density distribution of the compounds appeared concentrated between COCONUT and FooDB, in association with the large (98%) overlap between FooDB and COCONUT compounds (vide supra, Figure 1); a lower density was evidenced for DCM, CAS, and 3CLP. Figure 9 shows that the chemical space of the fragments was mostly defined by COCONUT fragments. Nevertheless, FooDB fragments presented a lower density compared to FooDB compounds, whereas a higher density was found for DCM fragments and CAS fragments concentrating in the chemical space covered by COCONUT fragments.

**Figure 10:**
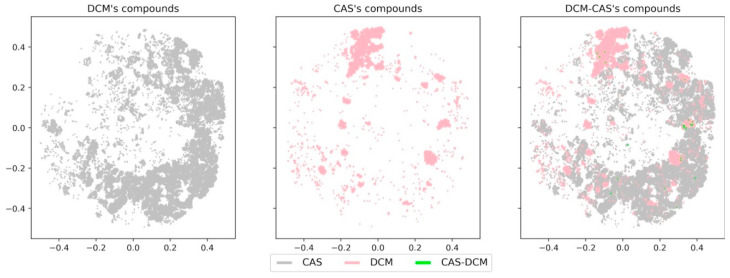
Visualization of the chemical space from CAS compounds (pink), DCM compounds (gray), and overlapping DCM-CAS compounds (green).

**Figure 11:**
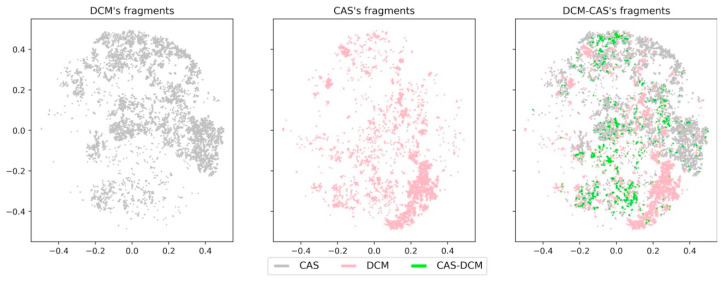
Visualization of the chemical space from CAS fragments (pink), DCM fragments (gray), and overlapping DCM-CAS fragments (green).

On the other hand, small molecules with scarce biological activity, like DCM, still converged in a large portion of chemical space covered by NPs (COCONUT) and CAS datasets. To further illustrate this point, Figure 10 and Figure 11 show a direct comparison of DCM, CAS, and the overlapping compounds and fragments. DCM compounds and CAS compounds hardly converged on chemical space, while CAS fragments and DCM fragments appeared to cover a large area of chemical space. For this reason, DCM fragments showed a significant larger overlap with CAS fragments in comparison with the original compounds. This observation suggests that fragments generated from DCM can be used as building blocks in de novo design of bioactive molecules, despite the source compounds’ lack of biological activity.

## 4. Conclusions

Herein, we generated, analyzed the composition, and made publicly available a fragment library obtained from an extensive collection of NP. The source compounds and fragment libraries were compared to herein assembled fragment libraries of compounds of interest in drug discovery, including molecules with significance in COVID-19 research. It was concluded that, in general, the fragments generated retained the structural characteristics of the source compounds (COCONUT, FooDB, CAS, DCM, and 3CLP). This analysis found that compounds from NP and food chemicals were structurally more diverse and complex than compounds from CAS, DCM, and 3CLP. Fragments generated from COCONUT and FooDB were more diverse than those from DCM and 3CLP and less diverse than those of the CAS fragments. It was also concluded that fragments from DCM overlapped with bioactive compounds like those of the CAS subset studied in this work. This reinforces previous observations of DCM as a source of building blocks for designing bioactive molecules. Similarly, fragments of NP from COCONUT and FooDB appear to be important and valuable building blocks for the future de novo design of bioactive compounds. The fragment libraries of the reference databases generated in this work and focused on COVID-19 research (CAS and 3CLP) can be used to identify novel compounds of medical interest and are not currently available in commercial libraries. The fragment libraries for COCONUT and FooDB and the reference libraries DCM, CAS, and 3CLP that we developed in this work are publicly available at https://doi.org/10.6084/m9.figshare.13064231.v1. 

## Figures and Tables

**Table 1:** Compound data sets analyzed in this work.

Dataset	Original Compounds	Processed Compounds	Generated Fragments	Reference
COCONUT	432,706	382,248	52,630	[16]
FooDB	23,883	21,319	3186	[17]
Dark Chemical Matter (DCM)	139,352	139,326	14,001	[13]
Chemical Abstract Service (CAS) set focused on COVID-19	48,876	44,692	8432	[18]
Inhibitors of the main protease of SARS-CoV-2 (3CLP)	280	256	108	[15]

COCONUT, COlleCtion of Open NatUral producTs, FooDB, Food Database (FooDB, COVID-19, coronavirus disease 2019, SARS-CoV-2, severe acute respiratory syndrome coronavirus 2.

**Table 2:** Summary of the structural composition of compounds from COCONUT, FooDB, and reference datasets ^a^.

Structural Feature	COCONUT	FooDB	DCM	CAS	3CLP
Carbon atoms	25.640	26.563	18.059	22.496	25.828
Oxygen atoms	6.167	7.343	3.252	5.773	4.922
Nitrogen atoms	1.445	0.668	2.859	4.157	3.582
Heavy atoms	33.611	34.942	25.139	33.535	35.352
Fraction of sp^3^ carbons	0.506	0.620	0.342	0.489	0.291
Fraction of chiral carbons	0.154	0.152	0.028	0.145	0.069
Rings	3.962	2.243	2.881	3.628	3.617
Aliphatic rings	2.250	1.426	0.791	1.372	0.645
Aromatic rings	1.712	0.817	2.089	2.256	2.973
Heterocycles	1.711	1.020	1.408	2.056	1.500
Aliphatic heterocycles	1.166	0.770	0.619	0.865	0.363
Aromatic heterocycles	1.712	0.817	2.089	2.256	2.973
Spiro atoms	0.167	0.051	0.018	0.019	0.000
Bridgehead atoms	0.493	0.137	0.056	0.254	0.023

^a^ Mean of the distribution.

**Table 3:** Summary of the structural composition of fragments from COCONUT, FooDB, CAS, DCM, and 3CLP and overlapping fragments ^a^.

Structural Feature	COCONUT	FooDB	DCM	CAS	3CLP	Overlapping Fragments
Carbon atoms	18.504	12.991	10.181	9.904	8.926	5.179
Oxygen atoms	3.524	3.173	1.748	3.678	1.556	1.107
Nitrogen atoms	0.795	0.394	1.475	0.883	0.713	0.107
Heavy atoms	23.034	16.760	14.057	15.532	11.537	6.464
Fraction of sp^3^ carbons	0.557	0.615	0.330	0.656	0.298	0.318
Fraction of chiral carbons	0.189	0.199	0.054	0.240	0.071	0.062
Rings	2.999	1.739	1.686	1.496	1.398	0.571
Aliphatic rings	2.013	1.237	0.447	0.837	0.398	0.071
Aromatic rings	0.986	0.503	1.239	0.660	1.000	0.500
Heterocycles	1.087	0.577	0.899	0.787	0.574	0.179
Aliphatic heterocycles	0.751	0.390	0.313	0.573	0.176	0.036
Aromatic heterocycles	0.986	0.503	1.239	0.660	1.000	0.500
Spiro atoms	0.190	0.085	0.013	0.010	0.000	0.000
Bridgehead atoms	0.507	0.288	0.043	0.109	0.056	0.000

^a^ Mean of the distribution.

**Table 4:** Summary of the fingerprint-based structural diversity of the entire compounds.

Dataset	Morgan2 ^a^ (1024-bits)	MACCS Keys ^a^ (166-bits)
COCONUT	0.107	0.380
FooDB	0.092	0.322
DCM	0.136	0.407
CAS	0.117	0.473
3CLP inhibitors	0.127	0.403

^a^ Median similarity.

**Table 5:** Summary of the fingerprint-based structural diversity of the fragment datasets.

Dataset of Fragments	Morgan2 ^a^ (1024-bits)	MACCS Keys ^a^ (166-bits)
COCONUT	0.111	0.300
FooDB	0.106	0.241
DCM	0.125	0.243
CAS	0.095	0.222
3CLP inhibitors	0.147	0.214

^a^ Median similarity.
